# Breast and Tumour Volume Measurements in Breast Cancer Patients Using 3-D Automated Breast Volume Scanner Images

**DOI:** 10.1007/s00268-017-4432-6

**Published:** 2018-01-03

**Authors:** M. Lagendijk, E. L. Vos, K. P. Ramlakhan, C. Verhoef, A. H. J. Koning, W. van Lankeren, L. B. Koppert

**Affiliations:** 1 Department of Surgical Oncology, Erasmus MC Cancer Institute, Groene Hilledijk 301, 3075 EA Rotterdam, The Netherlands; 2 Department of Bio-informatics, Erasmus MC, ‘s-Gravendijkwal 230, 3015 CE Rotterdam, The Netherlands; 3 Department of Radiology, Erasmus MC, ‘s-Gravendijkwal 230, 3015 CE Rotterdam, The Netherlands; 4 Department of Surgical Oncology, Erasmus MC Cancer Institute, PO Box 5201, 3008 AE Rotterdam, The Netherlands

## Abstract

**Background:**

The resection volume in relation to the breast volume is known to influence cosmetic outcome following breast-conserving therapy. It was hypothesised that three-dimensional ultrasonography (3-D US) could be used to preoperatively assess breast and tumour volume and show high association with histopathological measurements.

**Methods:**

Breast volume by the 3D-US was compared to the water displacement method (WDM), mastectomy specimen weight, 3-D MRI and three different calculations for breast volume on mammography. Tumour volume by the 3-D US was compared to the histopathological tumour volume and 3-D MRI. Relatedness was based on the intraclass correlation coefficient (ICC) with corresponding 95% confidence interval (95% CI). Bland–Altman plots were used to graphically display the agreement for the different assessment techniques. All measurements were performed by one observer.

**Results:**

A total of 36 patients were included, 20 and 23 for the evaluation of breast and tumour volume (ductal invasive carcinomas), respectively. 3-D US breast volume showed ‘excellent’ association with WDM, ICC 0.92 [95% CI (0.80–0.97)]. 3-D US tumour volume showed a ‘excellent’ association with histopathological tumour volume, ICC 0.78 [95% CI (0.55–0.91)]. Bland–Altman plots showed an increased overestimation in lager tumour volumes measured by 3-D MRI compared to histopathological volume.

**Conclusions:**

3-D US showed a high association with gold standard WDM for the preoperative assessment of breast volume and the histopathological measurement of tumour volume. 3-D US is an patient-friendly preoperative available technique to calculate both breast volume and tumour volume. Volume measurements are promising in outcome prediction of intended breast-conserving treatment.

## Introduction

For early stage breast cancer, similar survival rates are obtained when performing a mastectomy or breast-conserving therapy (i.e. partial removal of the breast followed by whole breast irradiation; BCT) [1]. Considering the high survival rates [2], (surgical) treatment decisions should focus on health-related quality of life in addition to the oncological outcomes. The type of surgery performed influences health-related quality of life [3]. In order to improve cosmetic outcome following BCT, multiple studies have focused on (preoperative) radiological imaging to predict or improve the cosmetic outcome [4, 5].

One of these preoperative parameters is breast volume, commonly assessed in the area of breast reconstructive surgery [6]. Preoperative breast volume measurements have been described using various three-dimensional (3-D) techniques [6–10]. These techniques showed high concordance for the preoperatively accessed breast volume in comparison to the water displacement method (WDM or Archimedes’ method). The WDM is considered the gold standard for breast volume measurement, but is only available following resection [9, 11].

Tumour volume studied as preoperative parameter has been described to predict the expected resection volume [11–13]. The resection volume in BCT is known to influence cosmetic outcome [13–16]. Tumour volume measurement can be performed on both mammography and breast ultrasonography [11–13]. No gold standard is available for the preoperative assessment of tumour volume. In the postoperative setting the gold standard for tumor volume is the volume as based on the freshly excised tissue.

The tumour volume-to-breast volume ratio in combination with the quadrant of the breast where the tumour is located is expected to be predictive for the cosmetic outcome following BCT [5]. A precise measurement of both tumour and breast volume is needed to enable this preoperative prediction of the expected cosmetic outcome following BCT. To access these volumes, a ultrasonography was chosen since it has several advantages over the use of other radiological modalities: it is widely available, affordable, non-invasive and does not depend on ionising radiation as compared to a mammography. It was hypothesised that 3-D US could be used to measure breast and tumour volume and furthermore shows a good association with histopathological volumes. For this the ultrasound volume was compared to the WDM, histopathological mastectomy specimen weight, 3-D MRI and mammography for breast volume and the histopathological tumour volume, 3-D MRI and mammography for tumour volume.

## Materials and methods

This prospective study was approved by the Ethics Committee of the Erasmus MC. Patients operated between March 2015 and December 2015 with a preoperative breast MRI were included prior to surgery after written informed consent was obtained. Since the study is considered an feasibility study, no power analysis was performed. Patients undergoing a mastectomy were eligible for breast and tumour volume measurement. Patients undergoing a prophylactic mastectomy were eligible for breast volume measurement where those scheduled for BCT were eligible for tumour volume measurement. All measurements were performed by one observer.

### Histopathological evaluation

Breast volume (*N* = 20) was measured on freshly excised breast specimen using two techniques. Primarily, the water displacement method (WDM) was used intraoperatively. WDM is based on Archimedes’ theory and considered gold standard [9, 11]. The mastectomy specimen was submerged into a graduated cup partly filled with water. The displaced water is than equal to the volume of the specimen. Second the breast volume was calculated by multiplying the specimen weight (gram) by the molecular weight, estimated to be 0.958 g/cm^3^ [17]. This molecular weight resembles the situation where the breast consists of 50% fatty tissue and 50% fibro-glandular tissue.

**Fig. 1:**
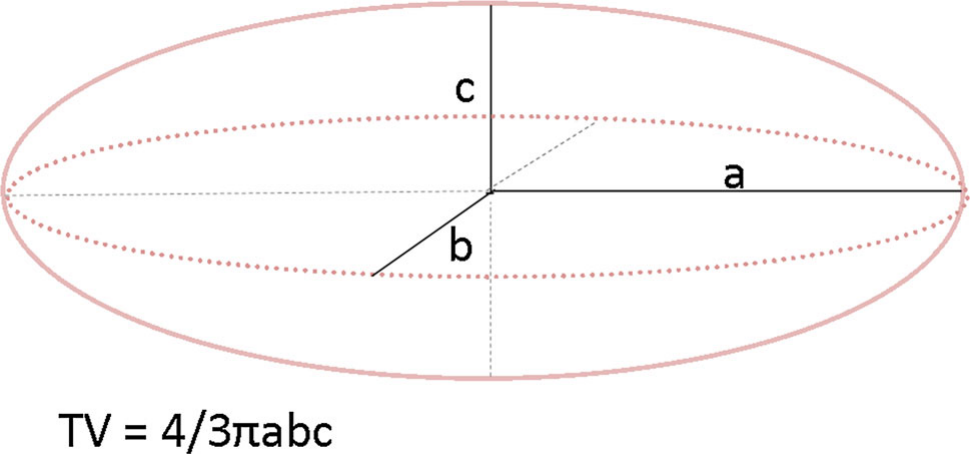
Mammographic determination of tumour volume [18]

Tumour volume (*N* = 23) was calculated assuming the tumour to resemble an obloid spheroid (Fig. 1) [18]. Three diameters of the carcinoma were obtained on the fresh tissue specimen. If ductal carcinoma in situ (DCIS) was present in the direct surrounding of the invasive component, the longest diameter of this area was additionally obtained. If a DCIS component was larger than 1.5 cm, patients were categorised as ‘DCIS > 1.5’. If a DCIS component was smaller than 1.5 cm, patients were categorised as ‘DCIS < 1.5’.

### Preoperative imaging

#### Automated breast volume scanner (ABVS) (3-D US)

3-D US was performed using the Siemens Automated Breast Volume Scanner (ABVS—ACUSON S2000™ ABVS, Siemens Medical Solutions, Inc, Mountain View, CA) [19]. The ABVS uses a linear transducer (17 cm) that automatically scans the breast in 60 s. Total breast volume was captured conducting three or five scans per breast based on size of the breast (i.e. anterior–posterior, lateral and medial or anterior–posterior, upper-lateral, lower-lateral, upper-medial and lower-medial). Ultrasonography data were analysed using a virtual reality desktop system developed by the department of Bioinformatics, Erasmus MC, running the V-Scope software [20]. This software enables volume measurements in a 3-D-plane by displaying the ABVS data on a virtual reality desktop system. Data can then be manipulated with a 3-D-mouse and wireless pointer. Calculations were based on differences found in grey levels (echogenicity).

#### 3-D breast MRI

Contrast enhanced-MRI data were analysed using the V-Scope software in a four-walled CAVE Automatic Virtual Environment I-Space system (Barco NV, Kortrijk, Belgium). Here eight projectors create an interactive hologram enabling manipulations with a wireless joystick. Volumes were calculated based on differences in grey levels representing different anatomical structures [21].

#### Mammography

**Fig. 2:**
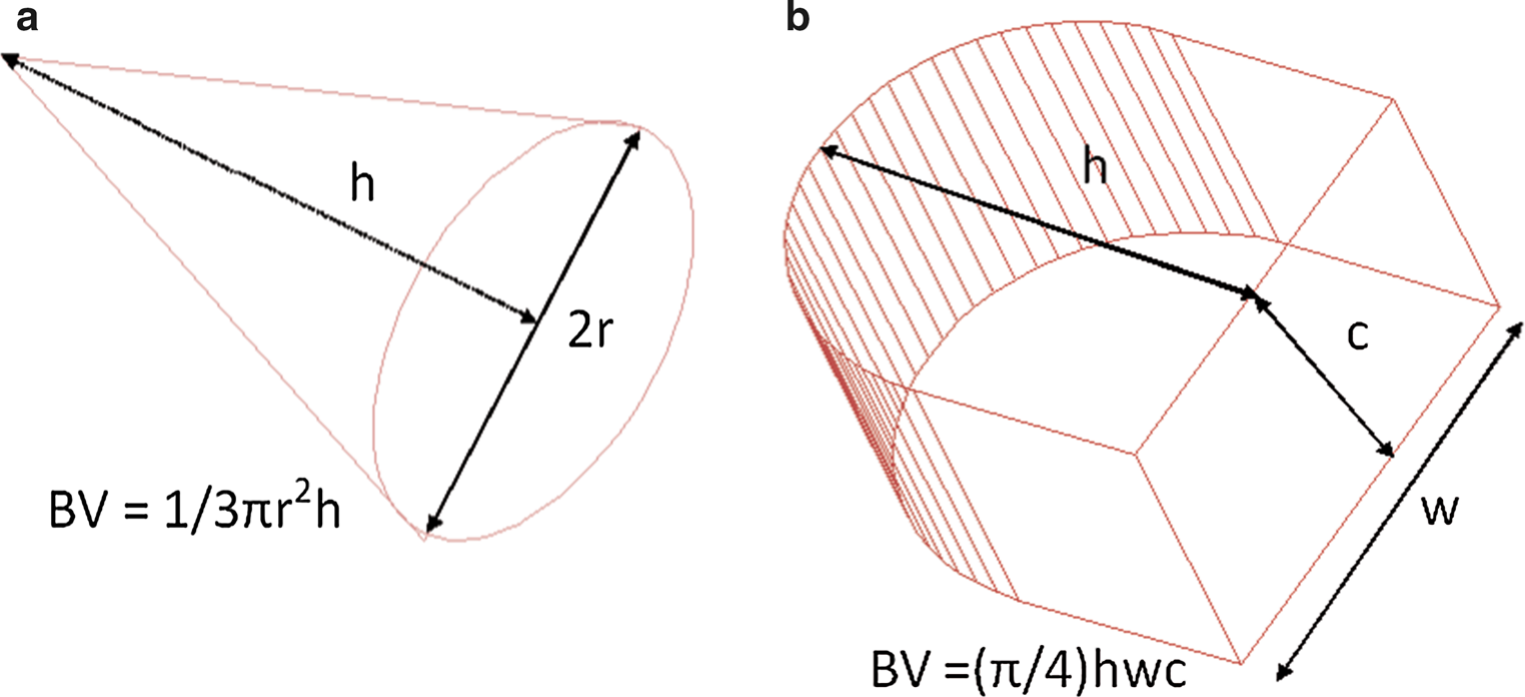
Mammographic determination of breast volume. **a** Breast volume as a elliptic shape [22]. **b** Breast volume as a circular cone [15, 17, 23]

Breast volume by mammography was measured based on two formulas (see below). The first equation considers the breast as a half-elliptic shape and accounts for the compression force of the breast (Fig. 2a) [22]. The height (*h*) and width (*w*) of the base of the breast were measured in a medio-lateral-oblique view of the mammography. The compression during the mammography was encountered in the formula as ‘c’, which is expressed as the compression in millimetres. The second measurement considers the breast to best resemble a circular cone (Fig. 2b). The height of the breast was expressed as ‘h’, and the width of the base of the breast was expressed as ‘*r*’. In literature, available different mammography views (i.e. cranio-caudal [15], medio-lateral-oblique [23] or a combination of the two [17]) are used for this second formula.

1 $${\text{Breast}}\,{\text{volume}}\,{\text{half - elliptic}}\,{\text{shape }} = \, \left( {\pi /4} \right)\,hwc$$

2 $${\text{Breast}}\,{\text{volume}}\,{\text{circular}}\,{\text{cone}} = 1/3\,\pi r^{2} h$$

Tumour volume was measured considering the tumour as an obloid spheroid equal to the tumour volume measured during histopathological evaluation (Fig. 1) [18].

### Data analysis

Data were analysed using IBM SPSS Statistics (21.0.0.1). The median breast volume (cm^3^) and tumour volume (cm^3^) with corresponding interquartile ranges were obtained per modality. The single measure intraclass correlation coefficient (ICC) with 95% confidence interval was used to calculate the measure of reliability between the different measurement techniques. For the interpretation of the reliability, an ICC of <0.40 ‘Poor’, an ICC of 0.40–0.59 as ‘Fair’, an ICC of 0.60–0.74 as ‘Good’, an ICC of 0.74–1.00 as ‘Excellent’ [24]. All breast volume measurement was compared to the WDM (gold standard). For tumour volume, a comparison was made to the volume measured on freshly excised specimens. Bland–Altman plots were used to visualise the accuracy for the preoperative breast volume and tumour volume techniques compared to histopathological volume. The *y*-axis displays the absolute difference between the two techniques (technique *A* – *B*), and the *x*-axis displays the averaged volume of the two techniques (technique (*A* + *B*)/2). The corresponding limits of agreement are graphically displayed to evaluate the difference in relation to the breast or tumour volume (i.e. the upper and lower limit representing the boundaries of the 95% confidence interval).

## Results

A total of 20 and 23 specimens were used for the evaluation of breast and tumour volume, respectively. Median breast volume measured by WDM (gold standard) was 462 cm^3^ [interquartile range, IQR (300–850)] (Table 1). All carcinomas available in the study were ductal carcinomas. Median tumour volume measured by histopathological evaluation was 1.33 cm^3^ [IQR (0.42–3.28)] (Table 1).

**Table 1:** Median volume (cm^3^) (interquartile range)

Breast volume (n = 20) (cm^3^)	
Water displacement method (WDM)	462 (300–850)
Breast volume by molecular weight	432 (350–676)
3-D US	427 (315–779)
3-D MRI	550 (436–1175)
MxKalbhen	575 (438–681)
MxCochrane	809 (706–1019)
MxFung	766 (614–1000)
MxKatariya	742 (559–1000)
Tumour volume (n = 23) (cm^3^)	
Histopathological tumour volume	1.33 (0.42–3.28)
3-D US	1.15 (0.43–1.79)
3-D MRI	2.24 (0.97–3.97)

*US* ultrasound, *Mx* mammography

### Breast volume

**Fig. 3:**
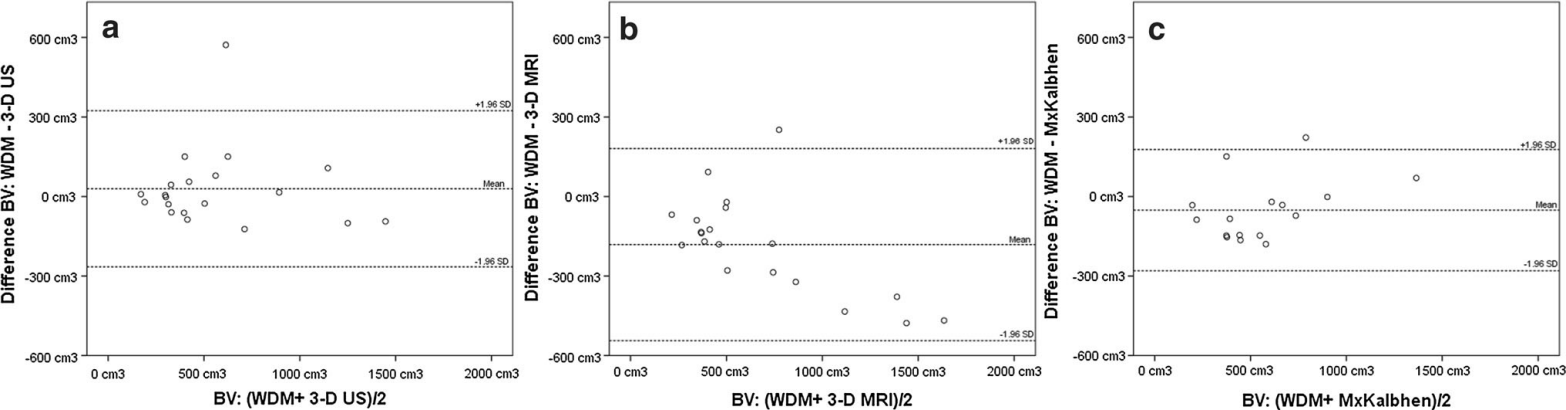
Bland–Altman plots for breast volume with the mean difference (solid line) and limits of agreement (dotted line). BV = breast volume, WDM = water displacement method, US = ultrasound, Mx = mammography. **a** Mean difference (WDM − 3-D US) as a function of the volume ((WDM + 3-D US)/2). **b** Mean difference (WDM − 3-D MRI) as a function of the volume (WDM + 3-D MRI). **c** Mean difference (WDM − MxKalbhen) as a function of the volume ((WDM + MxKalbhen)/2)

3-D US showed an ‘excellent’ association with the WDM, intraclass correlation coefficient (ICC) 0.92 [95% CI (0.80–0.97)] (Table 2). 3-D MRI, mammographic breast volume by Kalbhen, Katariya, Fung and Cochrane additionally showed an ‘excellent’ association with the WDM, ICC 0.95 [95% CI (0.87–0.98)], 0.91 [95% CI (0.77–0.97)], 0.90 [95% CI (0.75–0.96)] and 0.81 [95% CI (0.55–0.93)], respectively (Table 2). Agreements for WDM with 3-D US, 3-D MRI and mammographic breast volume by Kalbhen (MxKalbhen) are graphically displayed by Bland–Altman plots (Fig. 3). It is shown that the differences for the two techniques fall mainly between the limits of agreement. For the 3-D MRI, a substantial increase in the overestimation is seen with an increasing breast volume (Fig. 3b).

**Table 2:** Intraclass correlation coefficient^a^ (95% confidence interval) for breast volume measurements

	WDM	Molecular weight	3-D US	3-D MRI	MxKalbhen	MxKatariya	MxFung
Molecular weight	0.95 (0.87–0.98)						
3-D US	0.92 (0.80–0.97)	0.96 (0.91–0.99)					
3-D MRI	0.91 (0.78–0.96)	0.90 (0.76–0.96)	0.92 (0.81–0.97)				
MxKalbhen	0.92 (0.79–0.97)	0.94 (0.83–0.98)	0.90 (0.73–0.96)	0.86 (0.64–0.95)			
MxKatariya	0.91 (0.77–0.97)	0.94 (0.83–0.98)	0.92 (0.80–0.97)	0.94 (0.84–0.98)	0.88 (0.71–0.96)		
MxFung	0.90 (0.75–0.96)	0.96 (0.89–0.99)	0.93 (0.81–0.97)	0.94 (0.83–0.98)	0.94 (0.84–0.98)	0.95 (0.87–0.98)	
MxCochrane	0.81 (0.55–0.93)	0.88 (0.71–0.96)	0.84 (0.61–0.94)	0.85 (0.64–0.94)	0.89 (0.72–0.96)	0.83 (0.59–0.93)	0.95 (0.87–0.98)

*WDM* water displacement method, *US* ultrasound, *Mx* mammography, *Mx Kalbhen* mammographic breast volume calculated based on Kalbhen’s technique, *Mx Katariya* mammographic breast volume calculated based on Katariya’s technique, *MxFung* mammographic breast volume calculated based on Fung’s technique

^a^ICC of < 0.40 ‘Poor’, an ICC of 0.40–0.59 as ‘Fair’, an ICC of 0.60–0.74 as ‘Good’, an ICC of 0.74–1.00 as ‘Excellent’ [24]

### Tumour volume

**Fig. 4:**
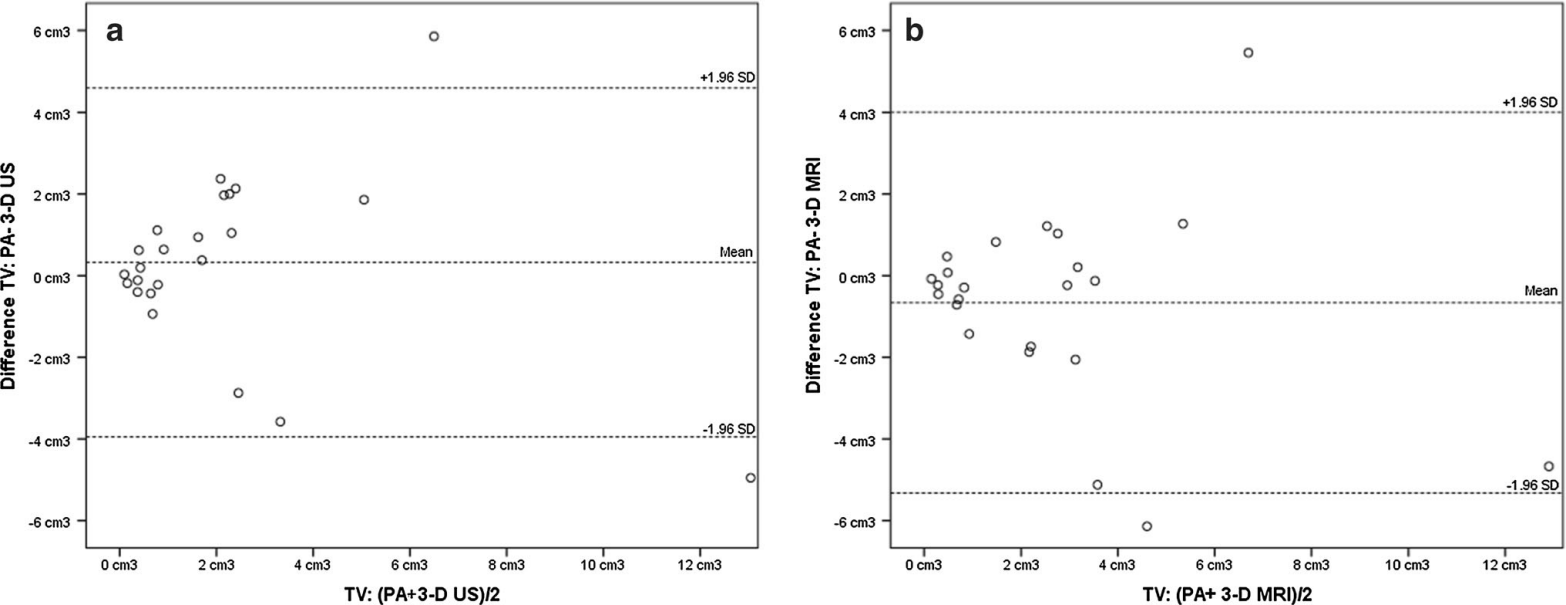
Bland–Altman plots tumour volume with the mean difference (solid line) and limits of agreement (dotted line). TV = tumour volume, US = ultrasound. **a** Mean difference (PA − 3-D US) as a function of the volume ((PA + 3-D US)/2). **b** Mean difference (PA − 3-D MRI) as a function of the volume ((PA + 3-D MRI)/2)

3-D US showed ‘excellent’ association with histopathological tumour volume, ICC 0.78 [95% CI (0.55–0.91)] (Table 3). 3-D MRI showed a ‘good’ association with histopathological tumour volume, ICC of 0.73 [95% CI (0.44–0.88)] (Table 3). Mammographic assessment of tumour volume was discarded since only in 14/23 (60.8%) tumour volume could be assessed. Agreements for histopathological tumour volume and 3-D US and 3-D MRI are graphically displayed by Bland–Altman plots (Fig. 4). Differences between the techniques fall within the limits of agreement except for one measurement.

**Table 3:** Intraclass correlation coefficient^a^ (95% confidence interval) for tumour volume measurements

	Histopathological tumour volume	3-D US
3-D US	0.78 (0.55–0.91)	
3-D MRI	0.73 (0.44–0.88)	0.94 (0.87–0.98)

*TV* tumour volume, *US* ultrasound

^a^ICC of < 0.40 ‘Poor’, an ICC of 0.40–0.59 as ‘Fair’, an ICC of 0.60–0.74 as ‘Good’, an ICC of 0.74–1.00 as ‘Excellent’ [24]

Ten patients (43.5%) had more than 1.5 cm diameter of ductal carcinoma in situ (DCIS) and were considered as ‘DCIS > 1.5’. For the ‘DCIS > 1.5’ group ‘Poor’ reliability scores were found for both 3-D US and 3-D MRI with histopathological tumour volume, ICC, respectively, 0.01 [95% CI (−0.64 to 0.63)] and 0.04 [95% CI (−0.61 to 0.66)]. For the ‘DCIS < 1.5’ group the association for 3-D US and 3-D MRI with histopathological tumour volume was ‘Excellent, ICC 0.86 [95% CI (0.57–0.96)] and ICC 0.88 [95% CI (0.63–0.96)], respectively.

## Discussion

Breast volume measurement by 3-D US as well as by Kalbhen mammography shows an ‘excellent’ association with gold standard water displacement method (WDM) with ICC of 0.92 and 0.95, respectively. Tumour volume measurement by 3-D US shows ‘excellent’ association with histopathological tumour volume (ICC 0.78). The importance of breast volume and tumour volume measurements preoperatively could be the cosmetic outcome prediction of breast-conserving treatment. In literature, volume measurements indeed enabled preoperative evaluation of the expected resection volume in ratio with the breast volume and thus a possible prediction of the expected cosmetic outcome [5, 13, 14]. Currently 3-D US is being used in a randomised controlled trial with the aim to preoperatively predict whether BCT will generate a good cosmetic result based on the tumour volume-to-breast volume ratio (NTR 4997).

A strength of the current study is that volumes were evaluated by all mentioned measurement techniques per patient: WDM and histopathological tumour volume if applicable, 3-D US, 3-D MRI, and tumour volume by mammographic formulas. To our knowledge, this is the first study to report on breast volume assessment using 3-D ABVS images. The availability of both breast volume and tumour volume measured on freshly excised specimens enabled an accurate comparison.

A limitation of the current study is that only ductal carcinomas of the breast were available within the cohort. It is therefore uncertain if results for tumour volume are generalisable for other histological subtypes. Mammography was considered unsuitable as a preoperative technique to access tumour volume at the time of evaluation; no tomography was available that could have possibly increased tumour visibility in dense breast tissue. The interpretation of the intraclass correlation coefficient (ICC) to rate the level of reliability varies in literature [11, 24, 25], making an unambiguous interpretation more difficult. Martins [25] suggested much higher cut-off values when interpreting the reliability of ultrasound in foetal measurements. Clauser et al. [11], however, used comparable cut-off values for their interpretation of the reliability of a 3-D US in breast cancer patients without referring to their guide for interpretation. Although different cut-off values are used, it should be taken into account that the ICC is dependent on the total variance found in the samples and should therefore be interpreted in the clinical setting used.

The precise differentiation between the invasive component and DCIS on histopathology enabled judgment on the performance of both 3-D US and 3-D MRI since DCIS is often not visible on ultrasonography as compared to the contrast enhanced 3-D MRI images [26, 27]. To evaluate the accuracy for both 3-D US and 3-D MRI, a differentiation was made in the histopathological evaluation for the invasive component (visible on ultrasonography) and for the amount of DCIS. A subgroup analysis, evaluating only patients without DCIS, was not performed due to a limited patient number (*n* = 7). It is however expected to show an ‘excellent’ association with tumor volume as seen in patients <1.5 cm DCIS. It is uncertain if the chosen differentiation between <1.5 cm DCIS and >1.5 cm DCIS is an accurate cut-off value which forms a limitation of the study. The preoperative calculation of the tumour volume in the presence of a known or expected large diameter of DCIS should be performed with caution.

Overall 3-D US enables an accurate preoperative, patient-friendly breast volume assessment without the use of ionising radiation as in mammography. As confirmed in our cohort, mammographic breast volume shows high relatedness with both the WDM [7] and breast volume by mastectomy specimen weight technique [17, 22]. As a preoperative technique, 3D-US is expected to be a suitable and patient-friendly alternative with equal high correlation to the WDM technique as obtained by mammography.

Tumour volume measured by ultrasound has been studied to preoperatively estimate the expected resection volume with high concordance to the histopathological volume [12, 13]. Clauser et al. [11] showed high concordance comparing tumour volume by MRI with tumour volume by hand-held ultrasonography and histopathological tumour volume. Various studies, however, showed an overestimation of the tumour volume by MRI [28–30]. This overestimation was confirmed within our cohort as presented by the Bland–Altman plot (Fig. 4) and can possibly be explained by the contrast enhancement images which colour the surrounding of the tumour or the presence of DCIS (as shown by the overall larger tumour volumes measured by MRI). As shown in the Bland–Altman analysis, 3-D US is more accurate in predicting histopathological tumour volume than 3-D MRI when smaller lesions are evaluated. As expected, 3-D MRI showed better relatedness to histopathological tumour volume in the presence of DCIS if <1.5 cm in the direct surrounding of the tumour (ICC 3-D MRI 0.88 compared to ICC 3-D US 0.86 both in relation to histopathological volume).

In conclusion, breast volume can accurately be assessed by mammography based on Kalbhen’s technique or by 3D-US which forms a more patient-friendly alternative. Tumour volume (with limited DCIS) measurement by 3D-US and 3D-MRI was comparably adequate with ‘excellent’ to ‘good’ relatedness for histopathology. Future research should further evaluate the use of preoperative volume measurements as a tool to predict cosmetic outcome of intended breast-conserving treatment. Currently a randomised controlled trial is ongoing evaluating the effectiveness of a preoperative prediction of the tumour volume-to-breast volume ratio to improve cosmetic outcome in breast cancer patients opting for BCT (NTR 4997).
